# Lower body mass index is associated with the achievement of target LDL in patients using PCSK9 inhibitors in Taiwan

**DOI:** 10.1186/s40001-025-02431-8

**Published:** 2025-03-15

**Authors:** Kuan-Chieh Tu, Wei-Ting Chang, Hui-Wen Lin, Po-Lin Lin, Yen-Wen Wu, Chao-Feng Lin, Hung-I. Yeh, Min-Ji Charng, Po-Hsun Huang, Tsung-Hsien Lin, Wei-Wen Lin, I.-Chang Hsieh, Feng-Yu Kuo, Ching-Pei Chen, Sheng-Hsiang Lin, Yi-Heng Li

**Affiliations:** 1 Division of Cardiology, Department of Internal Medicine, Chi Mei Medical Center, Tainan, Taiwan; 2 Department of Cardiology, Chi Mei Medical Center, Tainan, 710 Taiwan; 3 School of Medicine, College of Medicine, National Sun Yat-Sen University, Kaohsiung, 804 Taiwan; 4 Department of Public Health, College of Medicine, National Cheng Kung University, Tainan, Taiwan; 5 Department of Internal Medicine, College of Medicine, National Cheng Kung University Hospital, National Cheng Kung University, 138 Sheng Li Road, Tainan, Taiwan; 6 Cardiovascular Center, Department of Medical Research, MacKay Memorial Hospital and Department of Medicine, Mackay Medical College, New Taipei City, Taiwan; 7 Division of Cardiology, Cardiovascular Medical Center, Far Eastern Memorial Hospital, New Taipei City, Taiwan; 8 Department of Nuclear Medicine, Far Eastern Memorial Hospital, New Taipei City, Taiwan; 9 School of Medicine, National Yang Ming Chiao Tung University, Taipei, Taiwan; 10 Division of Cardiology, Department of Medicine, Taipei Veterans General Hospital, School of Medicine, National Yang Ming Chiao Tung University, Taipei, Taiwan; 11 Division of Cardiology, Department of Internal Medicine, Kaohsiung Medical University Hospital, Kaohsiung, Taiwan; 12 Department of Internal Medicine, Department of Pharmacology, Faculty of Medicine, College of Medicine, Kaohsiung Medical University, Kaohsiung, Taiwan; 13 Cardiovascular Center, Taichung Veterans General Hospital, Department of Life Science, Tunghai University, Taichung, Taiwan; 14 Division of Cardiology, Department of Internal Medicine, Chang Gung Memorial Hospital, Chang Gung University College of Medicine, Taoyuan, Taiwan; 15 Division of Cardiology, Department of Medicine, Kaohsiung Veterans General Hospital, Kaohsiung, Taiwan; 16 Division of Cardiology, Department of Internal Medicine, Changhua Christian Hospital, Changhua, Taiwan; 17 Biostatistics Consulting Center, College of Medicine, National Cheng Kung University Hospital, National Cheng Kung University, Tainan, Taiwan; 18 Institute of Clinical Medicine, College of Medicine, National Cheng Kung University, Tainan, Taiwan

**Keywords:** Statin, PCSK9 inhibitor, LDL-C target achiever, BMI

## Abstract

**Objective:**

Proprotein convertase subtilisin-kexin type 9 (PCSK9) inhibitors are a standard therapy for patients who respond poorly to or cannot tolerate statins. However, identifying responders to PCSK9 inhibitors remains unclear. This study investigates the characteristics of patients who achieve target LDL-C reduction (< 70 mg/dl) after PCSK9 inhibitor therapy.

**Methods:**

A multicenter, retrospective cohort study included patients initiating PCSK9 inhibitors at 11 teaching hospitals in Taiwan (2017–2021). Baseline characteristics, lipid-lowering therapies, and lipid profile changes were analyzed.

**Results:**

Among 211 patients (mean age 57.2 ± 13.1 years, 72.0% male), 73.5% used alirocumab and 26.5% used evolocumab. More than half had coronary artery disease and/or hypertension. Of these, 120 patients achieved the LDL-C target. Target achievers had a lower baseline BMI (25.8 ± 3.7 vs. 27.4 ± 4.5 kg/m^2^, P = 0.028) and a higher incidence of myocardial infarction and anti-platelet use compared to non-achievers. Baseline cholesterol and LDL-C levels were similar, but target achievers experienced greater LDL-C reductions (− 71.5; IQR − 81.8, − 62.2 vs. − 29.4; IQR − 38, − 10.5 mg/dl, P < 0.001), as well as decreases in triglycerides and increases in HDL-C. Glucose levels and liver enzymes did not differ significantly. Logistic regression revealed BMI as the only independent predictor of LDL-C target achievement (odds ratio: 0.899, 95% CI 0.821–0.984, P = 0.021).

**Conclusions:**

Lower BMI at baseline was associated with a higher likelihood of achieving LDL-C < 70 mg/dl after 12 weeks of PCSK9 inhibitor therapy. These findings support personalized strategies for optimizing cholesterol management in statin-intolerant patients while further investigations are required.

**Graphical abstract:**

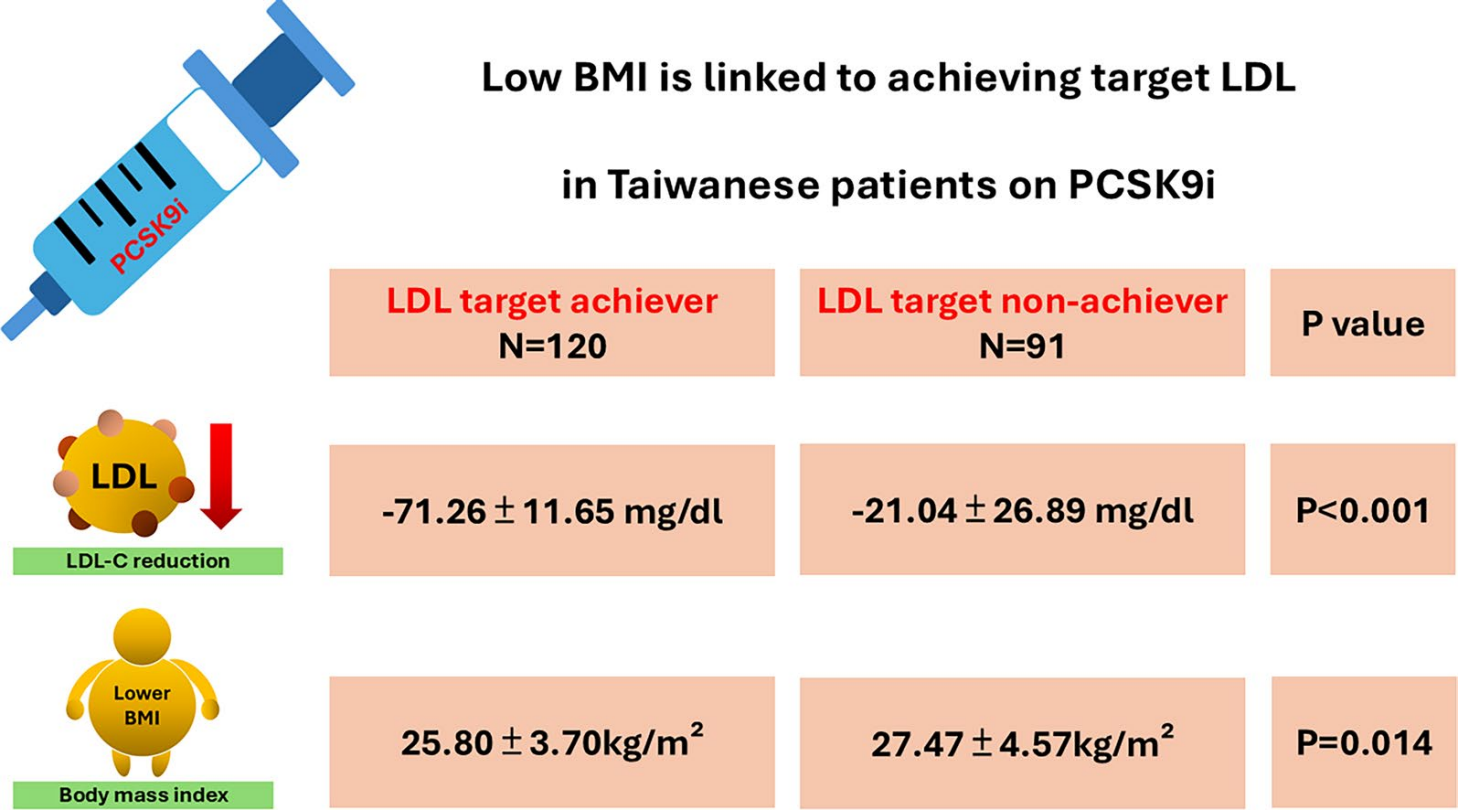

## Introduction

Atherosclerotic cardiovascular disease (ASCVD) is a leading cause of premature and preventable mortality globally [1]. Dyslipidemia, particularly elevated low-density lipoprotein cholesterol (LDL-C), plays a central role in the pathogenesis of atherosclerosis and is a key modifiable risk factor for ASCVD [2, 3]. The development of effective lipid-lowering therapies has significantly improved the management of dyslipidemia. Current clinical guidelines emphasize the importance of intensive LDL-C reduction in patients with ASCVD, often adopting the principle of “the lower, the better” as the cornerstone of dyslipidemia management [4–6]. Among lipid-lowering therapies, statins, which inhibit hepatic cholesterol production, and ezetimibe, which inhibits intestinal cholesterol absorption, have long been mainstays of treatment [7–9]. Recently, proprotein convertase subtilisin/kexin type 9 (PCSK9) inhibitors have emerged as a novel and highly effective class of agents for lowering serum lipid levels. PCSK9 is a serine protease that binds to the LDL receptor (LDLR), promoting its degradation and thereby reducing the liver’s ability to clear LDL-C from the bloodstream. PCSK9 inhibitors target this pathway through three primary mechanisms: (1) inhibition of PCSK9 binding to LDLR, (2) suppression of PCSK9 synthesis, and (3) inhibition of PCSK9 autocatalytic processing [10].

The most commonly used and commercially available PCSK9 inhibitors are monoclonal antibodies, which function by blocking the binding of PCSK9 to LDLR [11]. The efficacy of these agents, particularly evolocumab and alirocumab, has been demonstrated in major clinical trials, including the ODESSEY and FOURIER trials, which showed significant LDL-C reduction in patients with ASCVD [12, 13]. Currently, PCSK9 inhibitors are preferred for patients who fail to achieve adequate LDL-C reduction despite combination therapy with high-intensity statins (HIS) and ezetimibe or for those who are intolerant to statins [14]. Evidence suggests that individuals with documented ASCVD, or diabetes with target organ damage (TOD) may benefit most from PCSK9 inhibitors [15]. However, despite their efficacy, some patients exhibit suboptimal responses to PCSK9 inhibitors. Observational studies have reported that approximately 13% of patients are non-responders to PCSK9 inhibitors, a rate much higher than the less than 2% non-responders observed in clinical trials [16]. Furthermore, the high cost of PCSK9 inhibitors compared to other lipid-lowering agents emphasizes the importance to better identify the characteristics of patients who are likely to respond well to these therapies. Hence, this retrospective cohort study aims to identify the clinical characteristics of patients who achieve target LDL-C level following PCSK9 inhibitor therapy, providing insights into optimizing patient selection for this potent, yet costly, treatment option.

## Methods

### Study population

This multicenter, retrospective, cohort study was conducted from January 2017 to December 2021, gathering clinical and follow-up data from all patients who initiated PCSK9 inhibitor treatment at 11 major teaching hospitals in Taiwan. Patients were categorized into either the alirocumab or evolocumab group based on the initial treatment. Both alirocumab and evolocumab, available at similar prices, were used, with the choice of drug determined by the attending physician. All patients received at least two injections of the same drug post-initiation and remained on treatment for over 3 months. Patients who switched between alirocumab and evolocumab were categorized based on the drug used at the start of treatment. Those who died or were lost to follow-up were excluded from the study. Demographic information, vascular risk factors, disease history, and previous medications, including baseline lipid-lowering therapies, were collected using a predetermined protocol. Baseline therapies were classified as (1) high-intensity statins (HIS) (atorvastatin ≥ 40 mg/d or rosuvastatin ≥ 20 mg/d), (2) non-HIS (atorvastatin < 40 mg/d or rosuvastatin < 20 mg/d, or any other statin), or (3) statins plus ezetimibe (10 mg/d). Indications for PCSK9 inhibitor initiation and the presence of atherosclerotic cardiovascular disease (ASCVD)—such as coronary artery disease (CAD), myocardial infarction (MI), ischemic stroke/transient ischemic attack (TIA), or peripheral artery disease (PAD)—were documented. MI diagnosis was based on clinical presentation, electrocardiogram changes, and elevated cardiac biomarkers, following current guidelines. For patients undergoing coronary angiography and/or percutaneous coronary intervention (PCI), coronary atherosclerosis severity was categorized as 1-, 2-, or 3-vessel disease. Payers of PCSK9 inhibitors were classified as either Taiwan’s National Health Insurance (NHI) or non-NHI, depending on reimbursement or out-of-pocket/private insurance coverage. The follow-up duration was up to one year. The study adhered to the principles of the Declaration of Helsinki and was approved by the Institutional Review Boards of the 11 participating hospitals. All medical data were fully anonymized, and the study protocol was approved by the Medical Ethics Committee (IRB: A-ER-107-375) of National Cheng Kung University Hospital, Tainan, Taiwan. Given a retrospective study design, the inform consent was waivered.

### Outcome follow up

The primary outcome of the study was to assess the efficacy of LDL-C changes 12 weeks after initiating PCSK9 inhibitor therapy. Patients were regularly monitored by investigators at their respective hospitals. Lipid profiles, including total cholesterol, triglycerides, LDL-C, HDL-C, and other biochemical data, were collected at baseline and 12 weeks after starting PCSK9 inhibitors. LDL-C target achievers are defined as individuals with LDL-C levels < 70 mg/dL following the administration of a PCSK9 inhibitor, while those with LDL-C levels ≥ 70 mg/dL are classified as LDL-C target non-achievers.

### Statistical analysis

Data were presented as continuous variables with means ± standard deviations and categorical variables with numbers and percentages. If the normality assumption was violated, continuous variables were reported as medians with interquartile ranges (IQR). For comparisons of categorical variables between groups, the chi-square test was used if numbers were larger than 5; otherwise, Fisher’s exact test was applied. For continuous variables, Student’s t-test was used when the data followed a normal distribution; otherwise, the Mann–Whitney U test was conducted. Logistic regression was utilized to estimate odds ratios (OR) for LDL target achievers in patients receiving PCSK9 inhibitors post 12 weeks. Parameters presented with statistically significant difference (P < 0.05) between groups as well as age and sex in Table 1 were included in Logistic regression analysis. Multivariable logistic regression was adjusted with age, sex, body mass index, history of myocardial infarction, anti-platelet drug uses. Also, to further test the quality of the multivariate logistic regression model, we applied Hosmer–Lemeshow (HL) Goodness-of-Fit Test and Omnibus Test and generally suggested that our model provides a good fit for the observed data. Significance was set at p < 0.05 (2-tailed). SAS statistical package (version 9.4 for Windows; SAS Institute, Cary, NC, USA) was used for all analyses.

**Table 1:** Baseline clinical characteristics between LDL-C target achievers and non-achievers

	Total N = 211		LDL-C target non-achiever N = 91		LDL-C target achiever N = 120		*P* value
Age (yrs) (Mean ± SD)	57.2 ± 13.1		56.1 ± 15.0		58.0 ± 11.5		0.429
[Median (IQR)]	59.0 (48.0, 66.0)		58.0 (47.0, 69.0)		59.0 (50.0, 66.0)		
Sex (male)	152	(72.0)	65	(71.4)	87	(72.5)	0.987
BMI (kg/m^2^) (Mean ± SD)	26.4 ± 4.1		27.4 ± 4.5		25.8 ± 3.7		0.028
[Median (IQR)]	26.2 (23.8, 28.4)		27.0 (24.6, 29.4)		25.4 (23.2, 28.2)		
Hypertension	126	(59.7)	51	(56.0)	75	(62.5)	0.421
Diabetes mellitus	73	(34.6)	35	(38.5)	38	(31.7)	0.378
Smoker	54	(25.6)	21	(23.1)	33	(27.5)	0.569
HFrEF	18	(8.5)	7	(7.7)	11	(9.2)	0.896
Coronary artery disease	126	(59.7)	48	(52.8)	78	(65.0)	0.098
Myocardial infarction	72	(34.1)	23	(25.3)	49	(40.8)	0.027
Peripheral artery disease	10	(4.7)	5	(5.5)	5	(4.2)	0.749
Ischemic stroke or TIA	13	(6.2)	5	(5.5)	8	(6.7)	0.951
CKD without dialysis	25	(11.8)	9	(9.9)	16	(13.3)	0.581
ESRD under dialysis	4	(1.9)	1	(1.1)	3	(2.5)	0.636
COPD	5	(2.4)	3	(3.3)	2	(1.7)	0.654
Liver cirrhosis	2	(1.0)	0	(0.0)	2	(1.7)	0.507
Cancer	11	(5.2)	2	(2.2)	9	(7.5)	0.120
Anti-platelet drugs	181	(85.8)	72	(79.1)	109	(90.8)	0.027
Beta blocker	102	(48.3)	40	(44.0)	62	(51.7)	0.332
RAS inhibitor	91	(43.1)	32	(35.2)	59	(49.2)	0.058
*Non HIS*	77	(36.5)	34	(37.4)	43	(35.8)	0.933
*HIS*	14	(6.6)	6	(6.6)	8	(6.7)	1.000
*Non HIS* + *Ezetimibe*	48	(22.8)	26	(28.6)	22	(18.3)	0.112
*HIS* + *Ezetimibe*	72	(34.1)	25	(27.5)	47	(39.2)	0.104
*Alirocumab*	155	(73.5)	73	(80.2)	82	(68.3)	0.075
*Evolocumab*	56	(26.5)	18	(19.8)	38	(31.7)	0.075

Continuous variables were analyzed using the student t or Mann–Whitney U test, while categorical variables were assessed using the chi-square test or Fisher’s exact test

BMI: body mass index; CKD: chronic kidney disease; COPD: chronic obstructive pulmonary disease; ESRD: end stage renal disease; IQR: interquartile range; HFrEF: heart failure with reduced ejection fraction; HIS: high intensity statins; LDL-C: low-density lipoprotein cholesterol; RAS inhibitor: renin-angiotensin system inhibitors; SD: standard deviation; TIA: transient ischemic attack

## Results

### Baseline clinical characteristics

From January 2017 to December 2021, a total of 211 patients were enrolled in this study, of whom 120 participants successfully achieved the LDL-C target **(**Table 1**)**. Among the study population, 72.0% were male, with a mean age of 57 years old. The mean BMI was 26.4 ± 4.1 kg/m^2^, and patients who did not achieve the LDL-C target had a significantly higher BMI compared to those who met the LDL-C target. In the LDL-C target achievement group, there was a higher proportion of myocardial infarction (40.8 vs. 25.2%, P = 0.027) and a greater use of antiplatelet agents (90.8 vs. 79.1%, P = 0.027) compared to those who did not achieve the LDL-C target. No statistically significant differences were observed between the two groups with regard to other ASCVD, including coronary artery disease, peripheral artery disease, or transient ischemic attack (TIA)/stroke. The rate of other comorbidities, such as hypertension, diabetes mellitus, heart failure with reduced ejection fraction (HFrEF), chronic obstructive pulmonary disease (COPD), chronic kidney disease (CKD), end-stage renal disease (ESRD), and cancer, was similar between the groups. Additionally, there was no significant difference in the use of HIS, HIS combined with Ezetimibe or PCSK9 inhibitors (alirocumab and evolocumab) between the LDL-C target achievers and non-achievers. Detailed baseline characteristics of both groups are summarized in Table 1.

### The lipid profile at baseline

The baseline lipid profile and other biochemical parameters of the study population, stratified by LDL-C target achievement status, are summarized in Table 2. Mean total cholesterol levels were similar between LDL-C target non-achievers and achievers, with values of 206.0 (IQR 170.0, 248.0) mg/dL and 216.0 (IQR 179.5, 263.0) mg/dL, respectively (P = 0.187). Similarly, there was no significant difference in mean LDL-C levels between non-achievers (135.0; IQR 108.0, 172.0 mg/dL) and achievers (147.0; IQR 110.0, 183.5 mg/dL) with a combined mean of 143.0 (IQR 108.0, 174.0) (P = 0.108). HDL-C levels showed no statistical difference between the two groups, with non-achievers having a mean HDL-C of 40.0 (IQR 35.9, 47.0) mg/dL, and achievers at 41.0 (IQR 36.3, 52.1) (P = 0.264). Triglyceride levels were also comparable between the groups, with non-achievers at 130.5 (IQR 98.0, 178.5) mg/dL and achievers at 150.0 (IQR 103.0, 213.0) mg/dL (P = 0.141). Fasting blood glucose levels, glycated hemoglobin (HbA1C), liver function tests and mean creatinine levels were measured, with no significant difference observed between non-achievers and achievers.

**Table 2:** The lipid profile and other biochemical data at baseline

Baseline	LDL target non-achiever	LDL target achiever	Total	*P* value
	N = 91	N = 120	N = 211	
Cholesterol (mg/dL)	206.0 (170.0, 248.0)	216.0 (179.5, 263.0)	212.0 (175.0, 252.0)	0.187
LDL-C (mg/dL)	135.0 (108.0, 172.0)	147.0 (110.0, 183.5)	143.0 (108.0, 174.0)	0.108
HDL-C (mg/dL)	40.0 (35.9, 47.0)	41.0 (36.3, 52.1)	41.0 (36.0, 50.0)	0.264
Triglyceride (mg/dL)	130.5 (98.0, 178.5)	150.0 (103.0, 213.0)	141.5 (99.0, 205.0)	0.141
Fasting sugar (mg/dL)	103.0 (94.0, 121.0)	106.5 (92.0, 124.0)	105.0 (94.0, 123.0)	0.666
HbA1C	6.0 (5.7, 6.8)	5.9 (5.5, 6.5)	6.0 (5.6, 6.7)	0.300
AST (U/L)	24.0 (20.0, 37.0)	27.0 (20.0, 35.0)	25.0 (20.0, 35.0)	0.842
ALT (U/L)	24.5 (17.0, 45.0)	29.0 (20.5, 40.0)	26.5 (19.0, 41.0)	0.326
Creatinine (mg/dL)	1.0 (0.8,1.1)	0.9 (0.8, 1.1)	0.9 (0.8, 1.1)	0.655

AST: aspartate aminotransferase; ALT: alanine aminotransferase; HDL-C: high-density lipoprotein cholesterol; HbA1C: glycated hemoglobin; LDL-C: low-density lipoprotein cholesterol

### Changes of lipid profile at 12 weeks after initiating PCSK9 inhibitors

The improvement in lipid profile after 12 weeks of PCSK9 inhibitor therapy, stratified by LDL-C target achievement status, is summarized in Table 3. In the LDL-C target non-achiever group, total cholesterol decreased by − 20.4 (IQR − 28.4, − 6.4) mg/dL, whereas a more pronounced reduction of − 50.9 (IQR − 57.7, − 42.3) mg/dL was observed in the achiever group, with a statistically significant difference between the groups (P < 0.001). A similar trend was noted for LDL-C levels, with a decline of -29.4 (IQR − 38, − 10.5) mg/dL in the non-achiever group and a greater reduction of − 71.5 (IQR − 81.8, − 62.2) mg/dL in the achiever group (P < 0.001). Regarding triglyceride levels, slight increase of 0.8 (IQR − 24.1, 21.8) mg/dL was seen in the non-achiever group, whereas the achiever group experienced a decrease of − 30.8 (IQR − 42.9, − 7.9)mg/dL, with a significant difference between the two groups (P < 0.001). PCSK9 inhibitors also resulted in a more substantial elevation of HDL-C levels in the achiever group compared to the non-achiever group of 5.9 (IQR − 3.2, 22.2) mg/dL vs. 2.3 (IQR − 5.6, 10.3) mg/dL, P = 0.030). No statistically significant differences were observed between the two groups in other biochemical metrics, including fasting blood glucose, HbA1c, liver enzymes, and serum creatinine levels.

**Table 3:** Changes of lipid profile and other biochemical data at 12 weeks after initiating PCSK9 inhibitors treatment

	LDL target non-achiever	LDL target achiever	Total	*P* value
	N = 91	N = 120	N = 211	
Total cholesterol (mg/dL)	− 20.4 (− 28.4, − 6.4)	− 50.9 (− 57.7, − 42.3)	− 36.8 (− 52.9, − 22.2)	< 0.001
LDL-C (mg/dL)	− 29.4 (− 38, − 10.5)	− 71.5 (− 81.8, − 62.2)	− 54.7 (− 73.4, − 33.3)	< 0.001
HDL-C (mg/dL)	2.3 (− 5.6, 10.3)	5.9 (− 3.2, 22.2)	4.5 (− 4.5, 14.9)	0.030
Triglyceride (mg/dL)	0.8 (− 24.1, 21.8)	− 30.8 (− 42.9, − 7.9)	− 19.5 (− 40.3, 6.1)	< 0.001
Fasting sugar (mg/dL)	0.5 (− 9.1, 9.4)	− 1.0 (− 12.1, 8.8)	0.0 (− 10.3, 9.0)	0.585
HbA1C	0.0 (− 3.4, 1.8)	0.0 (− 3.2, 3.2)	0.0 (− 3.3, 1.9)	0.773
AST (U/L)	3.7 (− 10.0, 11.8)	0.0 (− 11.7, 18.0)	0.0 (− 10.3, 15.7)	0.551
ALT (U/L)	6.4 (− 17.6, 26.3)	− 5.9 (− 19.8, 14.5)	0.0 (− 18.6, 23.1)	0.064
Creatinine (mg/dL)	0.0 (− 10.0, 9.8)	− 3.6 (− 12.1, 7.1)	− 1.3 (− 10.9, 7.6)	0.102

Abbreviations as listed in Table 2

### LDL lowering effect among different starting dose of PCSK9 inhibitors

Table 4 summarizes the impact of different initial doses of PCSK9 inhibitors on LDL-C target achievement. In the non-achiever group, the majority of patients treated with either Alirocumab or Evolocumab were started on a PCSK9 inhibitor dose of 75 mg every 2 weeks (Q2W), whereas in the achiever group, most patients began with a dose of 140 mg Q2W. Among patients receiving alternative dosing regimens, among target achievers treated with Alirocumab, 15.9% of them received 140 mg Q3W to Q4W. Among target achievers treated with Evolocumab, 10.6% of them received 140 mg Q3W to Q4W or 420 mg Q4W. No statistically significant differences were observed between the groups in terms of initial dosing.

**Table 4:** The starting dose of PCSK9 inhibitors

Alirocumab				
LDL target non-achiever N = 73		LDL target achiever N = 82		*P* value
75 mg Q2W	62 (84.9)	140 mg Q2W	69 (84.2)	1.000
75 mg Q3W to Q4W	9 (12.3)	140 mg Q3W to Q4W	13 (15.9)	0.691
150 mg Q4W	0 (0.0)	420 mg Q4W	0 (0.0)	0.000
*Others	2 (2.7)	Others	0 (0.0)	0.000

Evolocumab				
LDL target non-achiever N = 18		LDL target achiever N = 38		*P* value
75 mg Q2W	11 (61.1)	140 mg Q2W	29 (76.3)	0.390
75 mg Q3W to Q4W	3 (16.7)	140 mg Q3W to Q4W	2 (5.3)	0.314
140 mg Q4W	3 (3.3)	420 mg Q4W	2 (5.3)	0.314
*Others	1 (5.6)	Others	5 (13.2)	0.652

Q2W: every 2 weeks; Q3W: every 3 weeks; Q4W: every 4 weeks

^*^Others indicated irregular use without specific intervals

### Logistic regression on factors linked to LDL-C target achievement

Table 5 summarizes the findings of both univariate and multivariable logistic regression analyses assessing factors associated with achieving LDL-C target. BMI was inversely correlated with the likelihood of reaching LDL-C goals. In univariate analysis, the odds ratio (OR) for BMI was 0.904, with a 95% confidence interval (CI) ranging from 0.833 to 0.982 (P = 0.016). This significant association persisted in the multivariable analysis, where the OR was 0.899 (95% CI 0.821–0.984, P = 0.021). Clinical key parameters such as age and sex did not show significant correlations with achieving LDL-C targets while the OR was 1.011 (95% CI 0.990–1.033, P = 0.298) and 1.055 (95% CI 0.575–1.933, P = 0.863), respectively. A history of myocardial infarction was associated with an increased likelihood of meeting LDL-C targets. In univariate analysis, it yielded an OR of 2.040 (95% CI 1.123–3.705, P = 0.019). However, this association lost significance in the multivariable model, with an OR of 2.230 (95% CI 0.996–4.996, P = 0.051). Anti-platelet drug usage was also positively associated with the odds of achieving LDL-C targets in univariate analysis (OR of 2.615; 95% CI 1.175–5.820, P = 0.019); however, these associations were not evident in the multivariable model (OR of 2.864; 95% CI 0.865–9.486, P = 0.085).

**Table 5:** The univariate and multivariable logistic regression of LDL target achiever in patients receiving PCSK9 inhibitors post 12 weeks

	Univariate			Multivariable		
	OR	95% CI	P	OR	95% CI	P
Age	1.011	(0.990–1.033)	0.298	1.009	(0.981–1.039)	0.517
Sex (vs. female)	1.055	(0.575–1.933)	0.863	0.779	(0.334–1.820)	0.564
Body mass index	0.904	(0.833–0.982)	0.016	0.899	(0.821–0.984)	0.021
History of myocardial infarction	2.040	(1.123–3.705)	0.019	2.230	(0.996–4.996)	0.051
Anti-platelet drug use	2.615	(1.175–5.820)	0.019	2.864	(0.865–9.486)	0.085

Multivariable logistic regression was adjusted with all the parameters

## Discussion

In this multicenter, retrospective, cohort study involving 211 patients, we identified several factors associated with a good response to PCSK9 inhibitors. Specifically, lower BMI, a higher prevalence of cardiovascular conditions such as MI, prior use of antiplatelet agents were significantly linked to responders. After adjusting for potential confounders, lower BMI was independently associated with a favorable response to PCSK9 inhibitors (Graphic abstract). Responders demonstrated more pronounced reductions in LDL-C levels and exhibited greater improvements in other lipid parameters compared to non-responders.

A PCSK9 inhibitor responder is typically defined as a patient who exhibits a significant reduction in LDL-C levels following PCSK9 inhibitor therapy. In most studies, responders are classified as those achieving a target LDL-C level, generally below 70 mg/dL, within a specified period (e.g. 12 weeks) after initiating treatment. In the present study, lower BMI was associated with the achievement of LDL-C targets in patients treated with PCSK9 inhibitors. It is believed that multiple metabolic and genetic factors contribute to the variability in serum PCSK9 levels, which can vary by up to 100-fold in the general population [17]. Previous observational studies have shown that obesity is associated with elevated serum PCSK9 levels [18, 19], and rising circulating PCSK9 levels have been linked to a reduced response to PCSK9 inhibitors [16]. This may partially explain the observed association between lower BMI and a positive response to PCSK9 inhibitors in this study, although the exact mechanism underlying this relationship remains unclear. Another potential explanation is that individuals with higher BMI may experience a larger volume of distribution for drugs, leading to wider dispersion of the PCSK9 inhibitor throughout the body, potentially reducing its concentration at target sites [20]. Although our study demonstrated an association between higher BMI and non-response to PCSK9 inhibitors, a previous retrospective study found no significant difference in response between obese and non-obese populations [16]. These conflicting results may stem from differences in the definitions and characteristics of obesity and BMI, as well as variations in the criteria used to define non-responders. Larger, well-designed studies are needed to further elucidate this relationship.

PCSK9 inhibitors are frequently prescribed for patients whose lipid levels remain elevated despite treatment with statins and/or ezetimibe, or for those who are intolerant to other lipid-lowering therapies. Statins, as the cornerstone of dyslipidemia management, reduce circulating and intracellular cholesterol levels, but this reduction also leads to the upregulation of sterol regulatory element-binding protein 2 (SREBP-2) and PCSK9 [21–24]. This phenomenon, often referred to as the “statin escape phenomenon,” attenuates the lipid-lowering efficacy of statins. PCSK9 inhibitors, by counteracting this effect, provide an additive benefit when combined with statin therapy, creating a synergistic impact on LDL-C reduction [22, 25]^)^. Previous studies have demonstrated that high-intensity statins significantly increased serum PCSK9 levels by 34% to 47%, [21, 23, 24, 26]. Likewise, Ezetimibe inhibits cholesterol absorption in the intestine, resulting in upregulated LDL receptor expression on hepatocytes as the liver compensates for lower cholesterol availability [27]. When combined with PCSK9 inhibitors, this mechanism also leads to an additive effect in lowering LDL-C levels [28, 29]. Conversely, in this study we did not observe significant differences of background HIS or HIS combined with Ezetimibe uses among LDL target achievers and non-achievers after the treatment of PCKS9 inhibitors. However, given that the majority of the studies patients were already treated with statins with or without Ezetimibe at baseline, our findings, at least in part, also supported that instead of hinder, HIS and ezetimibe enhance the LDL-lowering efficacy of PCSK9 inhibitors [28, 29]. This combination is especially valuable for patients who need intensive LDL-C reduction beyond what statins and ezetimibe can achieve alone.

## Limitations

The present study has some limitations. One limitation of this study is the lack of documentation on common causes of non-response to PCSK9 inhibitors, such as poor adherence to therapy, improper administration techniques, dermatological factors affecting systemic absorption, and inappropriate antibody distribution through the lymphatic system. Additionally, there is a potential for selection bias due to the retrospective nature of this study. However, since this is a retrospective study, all patients receiving PCSK9 inhibitors were included without requiring consent, reducing the risk of bias related to patients' willingness to participate. Second, serum PCSK9 levels were not available, limiting our ability to investigate the underlying causes of unusual responses to PCSK9 inhibitors. Third, we did not categorize non-responders into more specific subgroups, such as delayed responders (those who take longer to achieve LDL-C targets), partial responders (those who reduce LDL-C by less than 30%), or those who experience a loss of response (initially achieving a ≥ 30% reduction in LDL-C, followed by a decline in efficacy). This lack of detailed classification may have restricted a more nuanced analysis of different patterns of PCSK9 inhibitor response. Forth, despite monitoring many factors, the statistical comparisons between groups were underpowered. To test the quality of the multivariate logistic regression model, we applied Hosmer–Lemeshow (HL) Goodness-of-Fit Test and Omnibus Test. The HL Goodness-of-Fit Test evaluates how well a logistic regression model fits the data by comparing observed and predicted probabilities. In this case, the HL test yielded a p-value of 0.901, indicating a good model fit. The Omnibus Test assesses the overall significance of the logistic regression model by testing whether all regression coefficients are zero. If at least one coefficient is nonzero, the model has predictive power. Here, the p-value is 0.005, which is below the 0.05 threshold, confirming that the model is statistically significant. Although comparing several factors may underpower the statistical analysis, the model applied in this study suggested that it provides a good fit for the observed data. Lastly, as this is a retrospective study, the inherent limitations of this design prevent us from establishing definitive causality.

## Conclusions

Patients with lower BMI as baseline lipid-lowering therapy were more likely to achieve LDL-C levels below 70 mg/dL after 12 weeks of treatment. These findings highlight the potential for more personalized treatment strategies in patients who struggle to meet cholesterol targets with statin therapy alone. However, in a retrospective study analyzing multiple factors may reduce the statistical power, the inherent limitations may prevent us from drawing definitive causal conclusions while more investigations are required.

## Data Availability

The original data will be available upon reasonable request to the corresponding author.

## Abbreviations

ASCVD: Atherosclerotic cardiovascular disease
PCSK9: Proprotein convertase subtilisin-kexin type 9
LDL-C: Low-density lipoprotein cholesterol
LDLR: Low-density lipoprotein receptor
HIS: High-intensity statins
TOD: Target organ damage
CAD: Coronary artery disease
MI: Myocardial infarction
TIA: Transient ischemic attack
PAD: Peripheral artery disease
PCI: Percutaneous coronary intervention
NHI: National Health Insurance
OR: Odds ratios
HFrEF: Heart failure with reduced ejection fraction
COPD: Chronic obstructive pulmonary disease
CKD: Chronic kidney disease
ESRD: End-stage renal disease
HbA1C: Glycated hemoglobin
